# Gastrointestinal Comorbidities Associated with Atopic Dermatitis—A Narrative Review

**DOI:** 10.3390/ijms25021194

**Published:** 2024-01-18

**Authors:** Weronika Zysk, Alicja Mesjasz, Magdalena Trzeciak, Andrea Horvath, Katarzyna Plata-Nazar

**Affiliations:** 1 Department of Dermatology, Venereology and Allergology, Faculty of Medicine, Medical University of Gdansk, 80-214 Gdansk, Poland; weronikazysk@gumed.edu.pl; 2 Dermatological Students Scientific Association, Department of Dermatology, Venereology and Allergology, Faculty of Medicine, Medical University of Gdansk, 80-214 Gdansk, Poland; alicja.mesjasz@gumed.edu.pl; 3 Department of Paedistrics, Medical University of Warsaw, Żwirki I Wigury 63a, 02-091 Warszawa, Poland; andrea.hania@gmail.com; 4 Department of Paediatrics, Gastroenterology, Allergology and Paediatric Nutrition, Faculty of Medicine, Medical University of Gdańsk, Nowe Ogrody 1-6, 80-803 Gdańsk, Poland; katarzyna.plata-nazar@gumed.edu.pl

**Keywords:** atopic dermatitis, gastrointestinal disease, comorbidity

## Abstract

The current understanding of atopic dermatitis (AD) seems to be extending beyond a skin-confined condition frequently associated with allergic comorbidities, as in a number of epidemiological studies, the prevalence rate of a range of illnesses has been determined to be greater in patients with AD, or inversely. In most cases, the reasons for this are vague. A subset of these conditions are gastrointestinal disorders, including food sensitization (FS) and food allergy (FA), eosinophilic esophagitis (EoE) (it is of mixed background, both IgE-dependent and independent), food protein-induced enterocolitis syndrome (FPIES) (it exemplifies an IgE-independent food allergy), Crohn’s disease (CD), colitis ulcerosa (CU), celiac disease, irritable bowel syndrome (IBS), and gastroesophageal reflux disease (GERD). In this review, we performed a comprehensive search of the literature using the PubMed database. We addressed the epidemiology of the increased co-occurrence of these diseases with AD and discussed potential causes for this subject. Multiple gastroenterological comorbidities appear to be more common in patients with AD, according to our review. The mechanisms that underlie this phenomenon are largely unknown, highlighting the need for further study in this field.

## 1. Introduction

Atopic dermatitis (AD) is a chronic inflammatory skin disorder affecting approximately 20% of children and 2–5% of adults worldwide [1]. Although AD can develop at any age, it usually appears in early childhood [2]. The clinical presentation of the disease is characterized by pruritus, dryness, erythema, papules, and exudative changes, the location of which varies depending on the patient’s age [3]. Acute AD is characterized by exudative erythema accompanied by oedema, vesicles, and excoriations [2]. These skin symptoms are predominantly observed in infants. Xerosis and lichenification are more prevalent in adolescence and childhood [2]. The complex and multifactorial pathophysiology of AD, which is not fully understood yet, includes epidermal barrier defects, genetic abnormalities, dysregulation of the immune response, environmental factors, and skin microbiota dysbiosis [3]. Emollients serve as the foundation of AD treatment [2]. In mild cases, topical corticosteroids or topical calcineurin inhibitors (TCI) may be administered. Severe cases of atopic dermatitis frequently necessitate systemic treatment with, for instance, dupilumab or cyclosporin [2].

The main players in AD are from the T helper 2 (Th2) immune axis. Furthermore, the altered immune response includes additional activation of Th1, Th17, and Th22 cytokine pathways depending on the phase of the disease, age, and ethnicity of the patient [4]. Acute lesions in AD result from the accumulation of cytokines of the Th2 and Th22, and to a lesser extent, Th17, axes. As AD lesions become chronic, significant increases in Th1 cytokines are observed, along with an intensification of Th2 and Th22 responses [4]. The dominance of various types of cytokines involved in AD development, varying by ethnicity, leads to different AD phenotypes and endotypes [4]. Asian AD is characterized by greater Th17 and lower Th1 axis activation compared to European American AD, which shows a more pronounced Th2, Th22, and Th1 immune response [4]. Activation of the Th2/Th22 axis is predominant in African-American AD [4]. According to the age of patients, in adults, the Th22, Th17, and Th1 pathways are involved, while pediatric patients are characterized by less Th1 activation [5]. Additionally, AD can be divided into extrinsic and intrinsic AD based on IgE levels. The traditional immune polarization towards Th2, elevated IgE levels, eosinophilia, and greater prevalence of food allergy are associated with the extrinsic phenotype (80%) [4,5]. Whereas AD patients with the intrinsic phenotype (20%) exhibit normal levels of IgE and greater immune polarization towards Th1 and Th17/Th22 [4,5].

AD is associated with numerous atopic comorbidities as well as nonatopic ones [6]. The mentioned AD phenotypes, endotypes, and factors contributing to the pathogenesis of this dermatosis may have importance in the increased coexistence of certain diseases within particular AD subgroups of patients. Additionally, certain AD medications may exacerbate the symptoms of comorbidities, whereas others may be effective in treating both conditions [7]. Gaining insight into the factors that contribute to this coexistence is not only scientifically significant but also facilitates the development of personalized treatments [7]. Addressing the increased occurrence of cardiovascular, autoimmune, neurological, psychiatric, ocular, and some neoplastic diseases and endocrine disorders identified in epidemiologic studies among AD patients is a significant challenge [7,8,9]. 

While the relationship between AD and atopic comorbidities such as allergic asthma, rhinitis, and conjunctivitis is well established, the co-existence of nonatopic diseases with AD has gained attention only in recent years [10]. The association between AD and multiple atopic and nonatopic diseases is unlikely to be explained by simple mechanisms but rather seems to be complex, multifactorial, and bidirectional [6,10].

**Figure 1:**
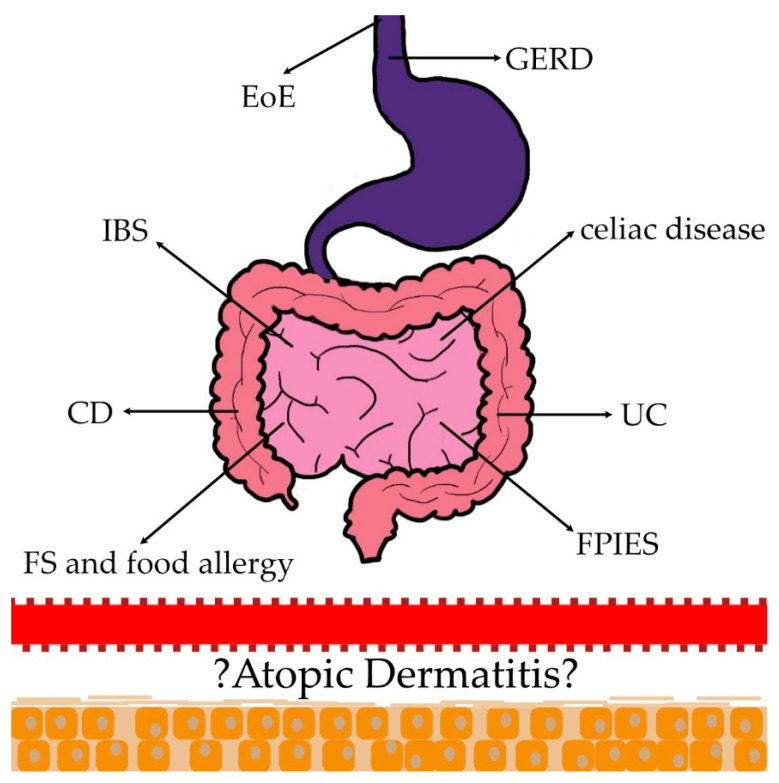
Several epidemiological studies demonstrate the association between atopic dermatitis (AD) and gastrointestinal comorbidities (food sensitization (FS) and food allergy (FA), eosinophilic esophagitis (EoE), food protein-induced enterocolitis syndrome (FPIES), Crohn’s disease (CD), colitis ulcerosa (CU), celiac disease, irritable bowel syndrome (IBS), gastroesophageal reflux disease (GERD)). The mechanisms are unlikely to be easily explicable but rather complex, multifactorial, and bidirectional.

Our objective is to provide a comprehensive summary of the gastrointestinal comorbidities of AD, including food sensitization (FS) and food allergy (FA), eosinophilic esophagitis (EoE), food protein-induced enterocolitis syndrome (FPIES), Crohn’s disease (CD), colitis ulcerosa (CU), celiac disease, irritable bowel syndrome (IBS), and gastroesophageal reflux disease (GERD) *(*Figure 1). This review paves the way for understanding the relationship between these gastrointestinal diseases and AD and sheds light on the need for further research in this area. 

## 2. Material and Methods

A comprehensive search of the literature using the PubMed (https://pubmed.ncbi.nlm.nih.gov/ (accessed on 8 February 2023)) electronic database using the search queries “(inflammatory bowel disease AND atopic dermatitis)”, “(Crohn’s disease AND atopic dermatitis)”, “(ulcerative colitis AND atopic dermatitis)”, and “(irritable bowel syndrome AND atopic dermatitis)” was performed in the third week of January 2023, from the database inception to the 22 January 2023. Further comprehensive research of the literature was conducted in the first week of February 2023, from the database inception to the 2 February 2023, using the queries: “(eosinophilic esophagitis AND atopic dermatitis)”, ”(food protein-induced enterocolitis syndrome AND atopic dermatitis)”, and “(celiac disease AND atopic dermatitis)”, “(food sensitization AND atopic dermatitis)” and (food allergy AND atopic dermatitis)”. The “(gastroesophageal reflux disease AND atopic dermatitis)’’ search was conducted in the second week of February 2023, from the database’s inception to the 8 February 2023. 

Non-related records, non-English manuscripts, personal opinions, duplicates, not relevant manuscripts, not originals, and not providing information concerning the earlier mentioned topics were excluded from the analysis.

## 3. Discussion

### 3.1. Food Sensitization (FS) and Food Allergy (FA)

The term food allergy (FA) is used to describe food hypersensitivity reactions mediated by immunologic mechanisms, which can be IgE-mediated or non–IgE-mediated. In turn, food sensitization (FS) means the production of food-allergen-specific IgE without presenting symptoms upon exposure to certain food allergens. In other words, FS is a prerequisite for IgE-mediated FA [11]. Thus, FS and FA are not synonymous.

Data from numerous studies indicate that the rates of FS are high in patients with AD, while the frequency of a challenge-proven FA may be lower. This is because not all sensitized patients develop symptoms upon exposure to the allergen [11,12]. For instance, the results from the DARC cohort have shown that up to 53% of children with AD were sensitized to food allergens (they had positive food-specific immunoglobulin E (IgE) and/or skin prick tests (SPTs)), while FA was confirmed on an oral food challenge in 15% of them [13]. The prevalence of FA in the general population is estimated at 0.1–6% [14], while in infants and young children with AD it is estimated at 37%, and among adults with AD it is estimated at 10% [15]. According to some scientific reports, the frequency of FA in AD patients is reported to be up to 80% [11].

There is a well-established association between AD, FS, and FA, especially in childhood [12]. Population-based studies have found that the likelihood of FS (i.e., the presence of food-specific IgE) in infants with AD is up to 6 times higher than in infants without AD (OR = 6.18, 95% CI 2.94–12.98, *p* < 0.001) [12]. Moreover, among 619 3-month-old exclusively breastfed infants, the association between AD and FS to milk, raw egg, cod, sesame, and peanut was significantly stronger in AD with a SCORAD > 20 (aOR 25.60, 95% CI 9.03–72.57, *p* < 0.001) than in AD with a SCORAD < 20 (aOR 3.91, 95% CI 1.70–9.00, *p* = 0.001) [16]. A large population-based study conducted in Australia has found that infants with AD are approximately 5 times more likely to develop IgE-mediated FA than infants without AD. Additionally, it has been shown that infants with AD are 6 and 11 times more likely to have egg and peanut allergies, respectively [17]. The risk of FA development is the greatest among infants with early-onset severe AD. In accordance with a study conducted by Martin et al., almost 50% of infants developing severe AD in the first 3 months of life develop classic FA by 1 year of age [17]. Another study has shown that the development of AD before the age of 1 year and persistent AD had the strongest associations with the occurrence of FA at the age of 2 years (OR 9.861, 95% Cl 9.115–10.668) and 3 years (OR 11.794, 95% Cl 10.721–12.975). In turn, AD onset at the age of 3 years had a weaker association with FA (OR 2.373, 95% Cl 2.02–2.789) [18]. Summing up, early-onset, severe, or persistent AD appears to be particularly associated with FA [12].

There is a significant body of evidence supporting the role of epicutaneous sensitization in the development of clinical FA. Results from the Avon Longitudinal Study of Parents and Children, including 13,971 preschool children, have shown that the use of peanut oil on inflamed skin was significantly associated with the occurrence of peanut allergy [19]. A theory known as the dual allergen exposure hypothesis suggests that early-life allergen exposure through the skin leads to FA development, whereas early oral exposure leads to tolerance [20]. The defect of the skin barrier, a hallmark of AD, may facilitate the penetration of environmental allergens, including food allergens, through the skin and initiate an inflammatory immune cascade of events leading to sensitization and food allergies [20].

**Figure 2:**
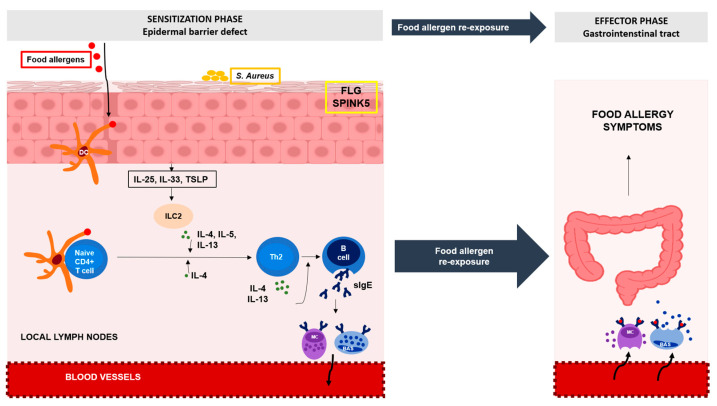
A disrupted skin barrier enhances the food allergens penetrating through the skin. Resident dendritic cell (DC) subsets capture these allergens and transport them to local lymph nodes, where they are processed and then presented to naive CD4+ T cells. Th2 (T helper cells 2) cytokine milieu favors the differentiation of naive CD4+ T cells into allergen-specific CD4+ T cells producing high levels of IL-4 and IL-13. IL-4 and IL-13 support the B cell isotype class switching to specific IgE (sIgE). sIgE bound to mast cells and basophils and primes these cells to react to future encounters with the allergen. Further exposure to the specific allergens leads to the degranulation of mast cells (MC) and basophils (BAS), resulting in FA symptoms. *Staphylococcus aureus,* through enhanced epidermal barrier damage, contributes to epicutaneous sensitization to food allergens. Mutations genes of *FLG* and *SPINK5* underlying skin barrier defects in AD have also been linked to FA. IL-25 (Interleukin 25), IL-33 (Interleukin 33), TSLP (Thymic stromal lymphopoietin), ILC2 (Innate lymphoid type-2-cells), IL-4 (Interleukin 4), IL-5 (Interleukin 5), IL-13 (Interleukin 13).

The immune response in IgE-mediated FA involves two stages—a sensitization phase and an effector phase (Figure 2). The allergic sensitization phase occurs in the setting of an impaired barrier, which promotes the release of proinflammatory epidermal cytokines, IL-25, IL-33, and thymic stromal lymphopoietin (TSLP), which initiate a type 2 inflammatory response. Following skin penetration of food allergens, epidermal Langerhans cells capture and process them and migrate to local lymph nodes, where processed allergens are presented to naïve CD4+ T cells. In the presence of IL-4 and endothelial-derived type 2 cytokines, these naive T cells differentiate into allergen-specific CD4+ T cells, producing high levels of IL-4 and IL-13. The presence of IL-4 and IL-13 promotes isotype switching in B cells to produce a large amount of IgE. The generation of allergen-specific IgE (sIgEs) created in this way binds via high-affinity FcεRI receptors to the mast cells and basophils and primes the cells to react on future encounters with the allergen, leading to sensitization of the individual to the allergen. During an effector phase, in sensitized people, further exposure to the specific allergens leads to cross-linking of IgE antibodies to FcεRI receptors and subsequent degranulation of mast cells and basophils, resulting in the development of FA symptoms [20].

Moreover, some genetic factors underlying skin barrier defects in AD have also been linked to FA. It has been demonstrated that mutations of FLG, which encodes filaggrin, and SPINK5, which encodes serine peptidase inhibitor Kazal type 5, are both independently associated with FA [21].

Skin microbiome dysbiosis is also believed to play a role in the development of epicutaneous sensitization and clinical FA. Staphylococcus aureus, which is more frequently present in AD skin than in healthy individuals through numerous mechanisms, enhances epidermal barrier damage and may thereby promote FA [22]. Indeed, higher rates of FA have been reported in children with AD colonized by Staphylococcus aureus compared to children without colonization by this pathogen [23].

### 3.2. Eosinophilic Esophagitis

Eosinophilic esophagitis (EoE) is a chronic, Th2-associated inflammatory disorder characterized by pronounced inflammation of the esophagus with cytokines such as Il-5, Il-13, Il-4, and IgE that contribute significantly to the EoE pathogenesis, similarly to AD [3,24]. In a mouse model, the greater concentration of IL-4 was insufficient to elicit esophageal inflammation, whereas that of IL-13 is satisfactory and essential for EoE-like inflammation in some instances [25]. Allergic etiology appears to be the underlying mechanism for EoE, as evidenced by the greater incidence of concomitant atopic disorders and frequent sensitization to various allergens in patients diagnosed with EoE [24]. Genetic and environmental components are also involved in the development of the condition [24].

In the meta-analysis summarizing the associations between atopic diseases and EoE, the incidence of AD in patients with EoE was higher compared to control subjects (OR 2.85, 95% CI, 1.87–4.34; *I*^2^ = 57.1%) [26]. Moreover, the meta-analysis indicated that the prevalence of all three disorders—asthma, allergic rhinitis (AR), and AD—is significantly higher in children and adults with EoE than in the general population [26]. Asthma, AR, AD, and food allergies each occurred at 44.7%, 27.1%, 25.2%, and 16.9%, respectively, in the US population, reaching up to 12 months after an EoE diagnosis [27]. In a study of 701 individuals, co-occurring allergy disorders were found in 91% of subjects with EoE [28]. Therefore, EoE is sometimes considered a late manifestation of the atopic march [28]. However, it is important to note that the legitimacy of the atopic march paradigm is currently being questioned [29].

An increase in the prevalence and incidence of allergic comorbidities or an increase in recognition may account for the rise in EoE over the last few years [30]. A greater number of biopsies are performed during endoscopy to test for this condition as a result of increased physician awareness [30]. EoE incidence peaks after that of AD, IgE-mediated food allergies, and asthma and coincides statistically with that of AR [31]. Exacerbations of EoE have been observed in patients with seasonal aeroallergen sensitization, and in the case of a food allergy, an elimination diet alleviated EoE symptoms, as it does in AD [32,33].

Susceptibility loci encoding TSLP, calpain 14 (CAPN14), and IL-4/kinesin family member 3A (IL4/KIF3A) were associated with a higher occurrence of AD in EoE patients [31,34]. TSLP is an epithelial cell-derived cytokine that is released at barrier surfaces in response to allergen exposure and has a role in the development and progression of type 2 inflammation [28]. TSLP has been reported to be overexpressed in esophageal biopsies from individuals with EoE, as well as in the skin biopsy of AD patients [28]. Loss-of-function mutations in the filaggrin (FLG) gene are present in EoE, resulting in impaired barrier function, which is similar to AD [24]. Additionally, patients with EoE exhibited decreased expression of E-cadherin, claudin, occludin, and desmoglein-1, which also contributes to barrier disruption [35]. 

Treatments for both EoE and AD appear to be relatively similar. Dupilumab, the most well-established biological drug for the treatment of AD, is also employed to treat EOE, as demonstrated by a phase 3 trial involving patients over the age of 12 [36]. Furthermore, topical glucocorticosteroids are used to treat both diseases [37]. 

### 3.3. Food Protein-Induced Enterocolitis Syndrome 

Food protein-induced enterocolitis syndrome (FPIES) is a non-IgE-mediated condition [38]. Recent studies have suggested a role of the innate immune system in FPIES, indicating the activation of monocytes, neutrophils, natural killer (NK) cells, and eosinophils in patients with FPIES following exposure to trigger foods; however, the underlying pathogenesis remains unknown [38]. The frequency of trigger food exposure affects the symptoms of FPIES, which are divided into acute and chronic [38]. In the majority of patients, FPIES manifests in infancy and self-resolves by school age [38]. The two most common allergens in children are cow’s milk and soy-based foods. Currently, there are no biomarkers or other diagnostic tests specific to FPIES; consequently, the diagnosis of chronic FPIES is based on clinical history [39].

As FPIES was given a separate disease code in 2015, epidemiological data are undoubtedly constrained [40]. Depending on the birth year, the incidence of pediatric FPIES appears to range between 0.17% and 0.42% [40]. The prevalence of AD (6–51%), IgE-FA (4–5%), asthma (3–25%), and AR (13.6–72%) has been consistently reported to be higher among children with FPIES compared to controls [40]. In the US study, 9.6% (5.8–15.5%) of children with parent-reported FPIES (*n* = 261) were diagnosed with AD [41]. However, in a different study involving 30 participants, AD was the most commonly occurring comorbid atopic disorder among FPIES patients, with a prevalence of 43.3% [42].

Elevated total serum IgE may be a laboratory marker indicating the potential future development of AD in toddlers with FPIES and normal skin, as FPIES typically manifests prior to the clinical manifestation of AD [43].

### 3.4. Crohn’s Disease (CD) and Ulcerative Colitis (UC)

Crohn’s disease (CD) and ulcerative colitis (UC) are examples of inflammatory bowel diseases (IBDs). Their development may be influenced by multiple factors, including genetics, gut microbiota dysbiosis (characterized primarily by decreased microbial diversity and reduced production of short-chain fatty acids [SCFAs]), environmental factors, and a mucosal immune imbalance. These factors are relevant to both CD and UC [44]. 

The most prominent immunological characteristic of CD is the excessive production of cytokines, such as IL-12 and IFN-α, which promote a Th1 phenotype as opposed to a Th2 one [45]. UC is believed to be primarily driven by Th2 cells [44]. Il-13, tumor necrosis factor (TNF)-α, which significantly reduces intestinal barrier resistance, and Il-23, Il-9, and Il-36 are the key cytokines engaged in the disease’s pathogenesis [44]. 

A retrospective UK cohort analysis that included children and adults with new-onset AD has shown a significantly increased risk for CD development in AD patients [46]. Similar results have also been found in a German cohort study [47]. Recently, a systematic review and meta-analysis were conducted, comparing 14 observational studies to assess the risk of autoimmune diseases in patients with AD [48]. Regarding CD, its increased prevalence in AD compared to healthy individuals has been established based on the pooling results of five studies (OR 1.66, Cl 1.50–1.84, *I*^2^ = 6.7%, *p* = 0.374) [48]. Furthermore, three cohort studies have revealed an increased risk of developing CD in AD patients (RR 1.38, 95% CI 1.17–1.63, *I*^2^ = 0.0%, *p* = 0.426) [48]. 

In the meta-analyses focusing on IBD, a bidirectional relationship between AD and IBD was identified (OR 1.35, 95% Cl 1.05–1.73, *I*^2^ = 89%; *p* < 0.01), and (OR 1.39, 95% CI 1.28–1.50, *I*^2^ = 89%; *p* < 0.01) [49]. Subgroup meta-analyses also revealed a bidirectional relationship between AD and UC (OR 1.23; 95% CI 1.11–1.35), or (OR 1.53; 95% CI 1.07–2.18). However, the risk of UC in patients with AD was not statistically significant (RR 1.11; 95% CI 0.88–1.44) [49]. Regarding CD, only a unidirectional relationship was shown. Patients with CD have increased odds of AD (OR 1.69; 95% CI 1.51–1.89), but the odds of CD in AD were not statistically significant (OR 1.14; 95% CI 0.60–2.15) [49]. In the meta-analysis by Lu et al. summarizing the incidence of AD and autoimmune diseases, the prevalence of UC was higher in AD compared with controls (OR 1.95, 95% CI 1.57–2.44, *I*^2^ = 67.2%, *p* = 0.009), and (RR 1.49, 95% CI 1.05–2.11, *I*^2^ = 40.2%, *p* = 0.196) [48]. Shi et al. also observed a bidirectional link between AD and UC in their meta-analysis (RR 1.48, 95% CI 1.06–2.04, *I*^2^ = 91%, *p* < 0.05), as well as AD and CD (RR 2.06, 95% CI 1.61–2.64, *p* < 0.01) [50]. However, not all single studies found positive associations between IBD and AD [51,52]. For instance, in a Taiwanese study, AD did not raise the risk of IBD [51].

One possible explanation for the relationship between CD and AD may be genetic. It has been established that AD and CD patients share genetic susceptibility loci such as 2q12.1, 11q13.5, 17q21.2, and 20q13.33 [47]. Similarly, three susceptibility loci, 4q27, 11q13.5, and 20q13.33, have been identified as common between AD and UC [47].

The important function of Th1 and Th17 lymphocytes has been confirmed in both CD and AD [3,45].

The unbalanced Th2 response and the release of proinflammatory cytokines lead to the impairment of the epidermal barrier as well as pruritus, whereas the upregulation of the Th2-mediated pathway leads to the disruption of epithelial tight junctions in UC [47]. There are some similarities in the specificity of the lesions, which are deep and transmural in CD but superficial in AD and UC [53]. 

The inability to maintain a homeostatic interaction with the microbiota appears to contribute to the pathophysiology of both IBD and AD [49]. Short-chain fatty acids (SCFA) are well-described metabolites obtained from the fermentation of dietary fiber by colonic bacteria [54]. However, their presence in the intestines is reduced due to the low fiber intake typical of a Westernized lifestyle. According to Trompette et al. [54], SCFA improves the integrity of the epidermal barrier, thereby limiting early allergen sensitization and potentially reducing the development of AD. The lack of SCFA may also contribute to the pathogenesis of IBD [54].

Recently, Kaempferol, a flavonoid identified in various dietary sources, has been reported to alleviate symptoms of AD via suppression of type 2 inflammation and improvement of barrier dysfunction by inhibition of TSLP expression and oxidative stress [55]. Furthermore, it has been demonstrated to prevent intestinal inflammation and modulate gut microbiota in mice fed a high-fat diet [56], which may link AD and IBD through the lens of dietary components and intestinal microbiota. It may be hypothesized that intestinal microbiota disorders can lead to an inappropriate immune response and inflammation in the gut, as well as systemically, potentially contributing to the development of both AD and IBD. However, current evidence is limited, so further research is needed to fully understand the complex relationship between these conditions.

### 3.5. Celiac Disease 

Celiac disease develops in genetically predisposed individuals who, in response to unidentified environmental variables, develop an immunological reaction that is subsequently triggered by gluten consumption [57]. Allotypes of HLA are required but not sufficient for disease development [58]. HLA-DQ2 and HLA-DQ8 are most strongly associated with celiac disease [58]. In celiac disease, the predominant immune response is Th1-related [59].

Meta-analysis revealed a greater incidence of celiac disease in AD versus controls (OR 1.98, 95% CI 1.51–2.60) [48]. In the study of 116,816 patients, AD was markedly linked to a higher rate of celiac disease (OR = 1.609, 95% confidence interval 1.42–1.82, *p* < 0.001) [59]. Men with AD had a greater correlation with celiac disease than women in a Swedish case-control study involving 104,832 individuals with AD and 1,022,435 controls [60]. In the UK study of 173,709 children, adults, and matched controls, the prevalence of celiac disease was higher in adults diagnosed with AD but not in children [46]. However, in the study conducted by Ress et al. [61], the prevalence of celiac disease was revealed to be four times higher in a group of 351 children with active AD compared to a randomly selected group of schoolchildren, as confirmed by serum testing (OR = 4.18, 95% CI 1.12–15.64) [61]. 

Damage to the intestinal barrier increases its permeability and may expose lymphoid tissue to a greater quantity of molecules, thereby provoking allergy symptoms at remote locations [59]. Additionally, AD is associated with an increase in FOXP3+ Tregs, whereas celiac disease is associated with a decrease in FOXP3+ Tregs [61]. Due to the enhanced permeability of the intestinal mucosa, the passage of (auto)antigens through the intestine may be facilitated. Consequently, FOXP3+ Tregs might be unable to suppress the inflammatory response, ultimately resulting in immune hyperactivation [61].

In addition, AD and celiac disease may share a similar genetic background, as polymorphisms of the CTLA4 gene have been linked to both conditions [59,61]. AD has also been associated with IgA deficiency, which in turn has been linked to an increased prevalence of autoimmune diseases [61]. 

### 3.6. Irritable Bowel Syndrome (IBS)

Irritable bowel syndrome (IBS) is a chronic gastrointestinal disorder characterized by recurrent abdominal pain or discomfort and altered bowel habits. The pathogenesis of the disease is still not fully understood. Factors thought to play a significant role in the development of IBS include psychological, immunologic, and genetic agents, chronic low-grade inflammation within the gut wall and the brain–gut axis, alteration of serotonin signaling, microbiota disorders, food sensitivity, and barrier dysfunction on mucosal surfaces [62].

A large retrospective observational study, including a Spanish children cohort, confirmed a significant association between AD and IBS (OR 1.90, 95% Cl 1.56–2.31, *p* < 0.001) [63]. Also, results obtained by Tsai et al. in their 8-year population-based cohort study suggest that children with AD are more likely to develop IBS [64]. AD children had a 1.45-fold greater risk (95%CI 1.32–1.59, *p* < 0.001) of developing IBS in comparison to the non-AD cohort. Additionally, it has been shown that girls and children aged ≥12 years have a greater risk of developing IBS [64]. The association between AD and IBS has also been found in adults. A cross-sectional study conducted in Turkey revealed that IBS was more common in the group with AD than in healthy individuals (56.9% vs. 28%, *p* < 0.001) [65]. Interestingly, patients with chronic pruritus have been reported to have a higher incidence of IBS than controls without chronic pruritus [66].

Immune responses may be one of the possible mechanisms linking IBS and AD. Mast cells (MC) are known to play a crucial role in allergic inflammation. In AD, MCs not only participate in type I hypersensitivity reactions associated with the presence of high-affinity IgE receptors (FcεRI) on their surfaces but also contribute to the development of pruritus and skin inflammation through the production of Th2 cytokines [67]. However, various mediators secreted by MC, such as tryptase, histamine, and prostaglandins, can also affect the gastrointestinal tract. In IBS, increased numbers of MC and their products have been described in the duodenal and colonic mucosa [68,69]. Further research has revealed that MC in patients with IBS contributes to increased intestinal permeability and the development of visceral hypersensitivity, causing symptoms such as abdominal pain and altered gastrointestinal motility [70].

The association between AD and IBS could also be explained at the genetic level. According to a study conducted by Camilleri et al., there are genetic variations that may affect local mucosal immune function in IBS, and some of them (*ORMDL3* and *c11orf30*) have also been associated with atopy [71]. 

An impaired epithelial barrier may be another possible mechanism linking AD with IBS. Several studies have identified the involvement of increased intestinal permeability and impaired tight junctions in the intestinal epithelium in the pathogenesis of IBS [67,68,72,73] . Jejunal biopsy specimens have shown lower expression of E-cadherin and zonula occludens (ZO)-1 in patients with IBS compared to healthy individuals [74]. Defects in E-cadherin and ZO-1 protein have also been found in AD patients, suggesting a possible link between AD and IBS mediated by an impaired epithelial barrier [75,76].

### 3.7. Gastroesophageal Reflux Disease (GERD)

Gastroesophageal reflux disease (GERD) is a common gastrointestinal motility disorder whose development may be influenced by factors such as increased body mass index, tobacco smoking, and genetic predisposition [77]. GERD is believed to be significantly associated with atopic diseases, particularly asthma [78,79,80]. 

Asthma and GERD are clinical conditions that often occur as comorbidities, and this relationship has been reported for many years [78]. Data on the association between AD and GERD have only recently emerged. An analysis conducted by Ahn et al., which aimed to assess the causality between GERD and atopic disorders, revealed that AD did not increase the risk of GERD development, but GERD increased the risk of AD occurrence. They found 21 genetic variants associated with GERD that also increased the risk of AD (OR, 1.21; 95% CI, 1.07–1.37; P_IVW_ = 3.32 × 10^−3^) [81]. The authors highlighted the existence of a complex genetic interplay between atopic diseases and GERD. In light of these results, they proposed that the predisposition to AD can arise from specific pathogenic mechanisms manifested by GERD [81]. Conversely, Brew et al. concluded that patients with AD are at risk of having GERD. In their large twin cohort, the odds of having GERD comorbidity in patients with AD were found to be OR 1.23 (95%CI 1.10–1.38) [82]. 

To date, only a few scientific reports have been published on the relationship between AD and GERD, which appear to be inconsistent. There is strong evidence of an association of AD with other atopic disorders, such as asthma and allergic rhinitis, whose relationship with GERD is relatively well established. This suggests a potential association between GERD and AD [6,78,79,80]. To explain this relationship, Brew et al., based on their analysis, have suggested that the coexistence of atopic diseases with GERD may be due to either genetic or common environmental factors. Furthermore, they proposed that a shared component involving affective traits, such as neuroticism, depression, or anxiety, may play a role. They implied that having an atopic disease may increase the risk of depression, anxiety, or neuroticism, which in turn triggers GERD [82]. Undoubtedly, further research is needed to establish the relationship between AD and GERD.

Table 1 summarizes the prevalence of certain gastrointestinal disease in patients with AD and vice versa.

### 3.8. Summary

The prevalence of vomiting, diarrhea, and regurgitation was higher among children diagnosed with AD [83]. The 4175-respondent study also found that adults with AD had a higher prevalence of self-reported, mild recurrent gastrointestinal symptoms than healthy controls [84]. Undoubtedly, upon close examination, these symptoms may indicate diseases that clinicians should be aware of and carefully investigate, especially with the knowledge that certain gastrointestinal comorbidities seem to be more common in AD patients in comparison to a healthy population. Clinicians may need to be aware of some epidemiological data, such as that FA is more commonly detected in infants with AD, whereas children whose parents have FPIES may have an increased risk of developing AD [41]. Our study provides a comprehensive overview of prevalent gastrointestinal disorders that have gained attention in recent years for their association with AD. It also includes epidemiological data for each disease and offers potential explanations based on current knowledge. We attempted to establish connections between the pathogenesis of the diseases and immunology, as this may be the determining factor in identifying common pathways and applying medicines that are effective in treating not one but two diseases. A case report highlights that the administration of upadacitinib at a dosage of 15 mg was an appropriate therapeutic choice for a 36-year-old male patient with both UC, a Th1-related disease, and AD, a Th2-mediated condition [85]. Both diseases were found to have a total and long-term clinical remission [85]. It is also noteworthy that the patient was initially classified as a low responder to dupilumab treatment [85]. We additionally looked for some other links, such as genetics, that may, in the future, to some extent, predict the disease onset. 

It is a well-known fact that patients with AD present differences in terms of gut microbiota in comparison to healthy individuals, and this can also be a subject of study and an explanation for the higher prevalence of certain diseases in AD patients or the other way around [86]. According to the review conducted by Pessoa et al., patients with AD have a higher prevalence of Clostridium difficile and Staphylococcus aureus in comparison to healthy individuals, contrary to reduced colonization of Bifidobacteria and Bacteroides when compared to healthy controls [86]. Additionally, it is worth mentioning that recent research on the role of dysbiosis in the gut microbiota in the pathogenesis and course of AD has made progress. Xue et al. conducted a two-sample Mendelian randomization study that indicates a possible causal connection between the number of microbes in the gut and the risk of AD and additionally indicates a genetic correlation between them [87]. In the study conducted by Liu et al., it was suggested that extrinsic and intrinsic AD are characterized by specific and different gut microbiomes [88]. This topic will undoubtedly continue to develop in the future.

The most important strength of this paper is providing comprehensive insight into the increased co-occurrence of gastrointestinal diseases with AD. The investigation of co-occurring non-atopic diseases with atopic dermatitis is still an area of ongoing research. It is only in recent years that more attention has been dedicated to exploring this aspect, which is very intriguing. Clinicians should be aware of this issue to personalize their approach for individual patients based on their unique characteristics and needs. Addressing comorbidities is crucial for comprehensive patient care, as they can impact overall health, quality of life, treatment decisions, and outcomes. Being aware of the comorbidities associated with AD has the potential to improve diagnosis, treatment, and management strategies. Additionally, the paper offers valuable insights into the potential mechanisms linking AD with gastrointestinal diseases, which may help clinicians better understand this phenomenon and provide the best care for individuals with AD.

The major limitation of the paper includes potential selection bias, as the selection of relevant studies to include in the analysis was done without a specified methodological plan. The selection process might have been influenced by subjective judgments, individual biases, or the availability of published research, which could lead to a skewed representation of the topic and potentially overlook important studies or perspectives.

## 4. Conclusions

According to the epidemiological studies cited above, multiple gastroenterological comorbidities appear to be more prevalent among patients with AD. The underlying mechanisms remain largely unexplored, highlighting the need for expanded research in this area. Analyzing the immunology of chronic inflammation and understanding how its correction, stimulation, or inhibition may help prevent the onset of a range of comorbidities is important for patients’ diagnosis, treatment, and care. As new AD treatment strategies are steadily introduced, their impact on AD-related comorbidities becomes an important area of study. Especially due to the lack of prevalent screening for comorbid conditions in AD patients, it is crucial for physicians to be aware of this issue to ensure heightened vigilance.

## Figures and Tables

**Table 1:** The prevalence of certain gastrointestinal disease in patients with AD and vice versa.

Gastrointestinal Disease	OR	RR	95% Cl	*p*-Value
The incidence of FS in AD infants [12]	6.18		2.94–12.98	*p* < 0.001
The incidence of FS to milk, raw egg, cod, sesame, and peanut in AD infants with a SCORAD > 20 [16]	25.60		9.03–72.57	*p* < 0.001
The incidence of FS to milk, raw egg, cod, sesame, and peanut in AD infant AD with a SCORAD < 20 [16]	3.91		1.70–9.00	*p* = 0.001
The incidence of AD in patients with EoE [26]	2.85		1.87–4.34	Not provided
The incidence of AD in patients with IBDs [49]	1.39		1.28–1.50	*p* < 0.01
The incidence of IBDs in patients with AD [49]	1.35		1.05–1.73	*p* < 0.01
The incidence of CD in AD patients [48]	1.66		1.50–1.84	*p* = 0.374
The incidence of CD in AD patients [48]		1.38	1.17–1.63	*p* = 0.426
The incidence of CD in patients with AD [49]	1.14		0.60–2.15	ns
The incidence of AD in patients with CD [50]		2.06	1.61–2.64	*p* < 0.01
The incidence of AD in patients with CD [49]	1.69		1.51–1.89	Not provided
The incidence of AD in patients with UC [49]	1.23		1.11–1.35	Not provided
The incidence of UC in patients with AD [49]	1.53		1.07–2.18	Not provided
The incidence of UC in patients with AD [49]		1.11	0.88–1.44	ns
The incidence of UC in patients with AD [48]	1.95		1.57–2.44	*p* = 0.009
The incidence of UC in patients with AD [48]		1.49	1.05–2.11	*p* = 0.196
The incidence of UC in patients with AD [50]		1.48	1.06–2.04	*p* < 0.05
The incidence of celiac disease in AD [48]	1.98		1.51–2.60	Not provided
The incidence of celiac disease in AD [59]	1.609		1.42–1.82	*p* < 0.001
The incidence of celiac disease in AD children [61]	4.18		1.12–15.64	Not provided
The incidence of IBS in AD children [63]	1.90		1.56–2.31	*p* < 0.001
The incidence of GERD in AD patients [82]	1.23		1.10–1.38	Not provided

Abbreviations: AD: atopic dermatitis; FS: food sensitization; EoE: eosinophilic esophagitis; IBDs: inflammatory bowel diseases; CD: Crohn’s disease; UC: ulcerative colitis; IBS: irritable bowel syndrome; GERD: gastroesophageal reflux disease; ns: non-significant.

## Data Availability

Not applicable.
